# Clinical spectrum of *STX1B*-related epileptic disorders

**DOI:** 10.1212/WNL.0000000000007089

**Published:** 2019-03-12

**Authors:** Stefan Wolking, Patrick May, Davide Mei, Rikke S. Møller, Simona Balestrini, Katherine L. Helbig, Cecilia Desmettre Altuzarra, Nicolas Chatron, Charu Kaiwar, Katharina Stöhr, Peter Widdess-Walsh, Bryce A. Mendelsohn, Adam Numis, Maria R. Cilio, Wim Van Paesschen, Lene L. Svendsen, Stephanie Oates, Elaine Hughes, Sushma Goyal, Kathleen Brown, Margarita Sifuentes Saenz, Thomas Dorn, Hiltrud Muhle, Alistair T. Pagnamenta, Dimitris V. Vavoulis, Samantha J.L. Knight, Jenny C. Taylor, Maria Paola Canevini, Francesca Darra, Ralitza H. Gavrilova, Zöe Powis, Shan Tang, Justus Marquetand, Martin Armstrong, Duncan McHale, Eric W. Klee, Gerhard J. Kluger, Daniel H. Lowenstein, Sarah Weckhuysen, Deb K. Pal, Ingo Helbig, Renzo Guerrini, Rhys H. Thomas, Mark I. Rees, Gaetan Lesca, Sanjay M. Sisodiya, Yvonne G. Weber, Dennis Lal, Carla Marini, Holger Lerche, Julian Schubert

**Affiliations:** From the University of Tübingen (S. Wolking, J.M., Y.G.W., H.L., J.S.), Department of Neurology and Epileptology, Hertie Institute for Clinical Brain Research, Tübingen, Germany; Luxembourg Centre for Systems Biomedicine (P.M.), University of Luxembourg, Esch-sur-Alzette; Pediatric Neurology and Neurogenetics Unit and Laboratories (D.M., R.G., C.M.), Children's Hospital Anna Meyer, University of Florence, Italy; Danish Epilepsy Centre (R.S.M.), Dianalund; Institute for Regional Health Services (R.S.M.), University of Southern Denmark, Odense; Department of Clinical and Experimental Epilepsy (S.B.), UCL Institute of Neurology and Epilepsy Society, UK, London; Division of Neurology (K.L.H., I.H.), Children's Hospital of Philadelphia, PA; Department of Pediatric Neurology (C.D.A.), Centre de Compétences Maladies Rares, CHU Besançon; Service de Génétique (N.C.), Hospices Civils des Lyon, Bron; GENDEV Team (N.C.), Neurosciences Research Center of Lyon, Bron, France; Neuropediatric Clinic and Clinic for Neurorehabilitation (K.S.), Epilepsy Center for Children and Adolescents, Schoen Klinik Vogtareuth, Germany; Beaumont Hospital (P.W.-W.), Dublin, Ireland; Department of Pediatrics, Division of Medical Genetics, Institute of Human Genetics (B.A.M.), Departments of Neurology and Pediatrics (A.N.), and Departments of Neurology and Pediatrics, and Institute of Human Genetics (M.R.C.), University of California, San Francisco; Department of Neurology (W.V.P.), University Hospitals Leuven, Belgium; Department of Pediatrics (L.L.S.), Hvidovre Hospital, Denmark; King's College Hospital (S.O., E.H., S.G., D.K.P.), London; Evelina London Children's Hospital (S.O., E.H., S.G.), London, UK; Section of Genetics (K.B., M.S.S.), Department of Pediatrics, University of Colorado and Children's Hospital Colorado, Aurora; Clinique Bernoise Montana (T.D.), Crans-Montana, Switzerland; Department of Neuropediatrics (H.M.), University Medical Center Schleswig-Holstein, Christian-Albrechts University, Kiel, Germany; National Institute for Health Research Oxford Biomedical Research Centre, Wellcome Centre for Human Genetics (A.T.P., S.J.L.K., J.C.T.) and Department of Oncology (D.V.V.), University of Oxford, UK; Epilepsy Center (M.P.C.), Health Sciences Department, San Paolo Hospital, University of Milan; Child Neuropsychiatry (F.D.), Department of Surgical Sciences, Dentistry, Gynecology and Pediatrics, University of Verona, Italy; Departments of Neurology and Clinical Genomics (R.H.G.) and Health Sciences Research and Clinical Genomics (E.W.K., C.K.), Mayo Clinic, Rochester, MN; Ambry Genetics (Z.P.), Aliso Viejo, CA; Department of Clinical Neuroscience (S.T.), King's College London; New Medicines (M.A., D.M.), UCB Pharma, Slough, UK; Neuropediatric Clinic and Clinic for Neurorehabilitation (G.J.K.), Epilepsy Center for Children and Adolescents, Schoen Klinik Vogtareuth, Germany; Research Institute for Rehabilitation, Transition and Palliation (G.J.K.), PMU Salzburg, Austria; Department of Neurology (D.H.L.), University of California, San Francisco; Neurogenetics Group (S. Weckhuysen), Center for Molecular Neurology, VIB, Antwerp; Laboratory of Neurogenetics (S. Weckhuysen), Institute Born-Bunge, University of Antwerp; Department of Neurology (S. Weckhuysen), Antwerp University Hospital, Antwerp, Belgium; Department of Basic & Clinical Neuroscience, Institute of Psychiatry, Psychology & Neuroscience (D.K.P.), MRC Centre for Neurodevelopmental Disorders (D.K.P.), King's College London, UK; Evelina London Children's Hospital (D.K.P.), London, UK; Department of Neuropediatrics (I.H.), University Medical Center Schleswig-Holstein, Christian-Albrechts University, Kiel, Germany; Institute of Neuroscience (R.H.T.), Henry Wellcome Building, Newcastle University; Neurology Research Group (M.I.R.), Institute of Life Science, Swansea University Medical School, Swansea, UK; Service de Génétique (G.L.), Hospices Civils des Lyon, Bron; GENDEV Team (G.L.), Neurosciences Research Center of Lyon, Bron, France; NIHR University College London Hospitals Biomedical Research Centre (S.M.S.), UCL Institute of Neurology, London, UK; Cologne Center for Genomics (D.L.), University of Cologne, Germany; Stanley Center for Psychiatric Research (D.L.) and Program in Medical and Population Genetics (D.L.), Broad Institute of MIT and Harvard, Cambridge; Psychiatric and Neurodevelopmental Genetics Unit (D.L.), Massachusetts General Hospital and Harvard Medical School, Boston.

## Abstract

**Objective:**

The aim of this study was to expand the spectrum of epilepsy syndromes related to *STX1B*, encoding the presynaptic protein syntaxin-1B, and establish genotype-phenotype correlations by identifying further disease-related variants.

**Methods:**

We used next-generation sequencing in the framework of research projects and diagnostic testing. Clinical data and EEGs were reviewed, including already published cases. To estimate the pathogenicity of the variants, we used established and newly developed in silico prediction tools.

**Results:**

We describe 17 new variants in *STX1B*, which are distributed across the whole gene. We discerned 4 different phenotypic groups across the newly identified and previously published patients (49 patients in 23 families): (1) 6 sporadic patients or families (31 affected individuals) with febrile and afebrile seizures with a benign course, generally good drug response, normal development, and without permanent neurologic deficits; (2) 2 patients with genetic generalized epilepsy without febrile seizures and cognitive deficits; (3) 13 patients or families with intractable seizures, developmental regression after seizure onset and additional neuropsychiatric symptoms; (4) 2 patients with focal epilepsy. More often, we found loss-of-function mutations in benign syndromes, whereas missense variants in the SNARE motif of syntaxin-1B were associated with more severe phenotypes.

**Conclusion:**

These data expand the genetic and phenotypic spectrum of *STX1B*-related epilepsies to a diverse range of epilepsies that span the International League Against Epilepsy classification. Variants in *STX1B* are protean and contribute to many different epilepsy phenotypes, similar to *SCN1A*, the most important gene associated with fever-associated epilepsies.

Genetic generalized epilepsies (GGEs) and genetic epilepsies with febrile seizures (FS) plus (GEFS+) are genetically and phenotypically heterogeneous epileptic disorders. GGEs share common clinical hallmarks such as seizure types, generalized epileptic discharges on EEG, and typical onset between childhood and adolescence. Most GGE cases have a polygenic inheritance, but a few monogenic causes have been identified, such as *GABRA1*,^1^ *SLC2A1*,^2^ *CACNA1H*,^3^ as well as microdeletions, such as 15q13.3.^4^ GEFS+ is a familial epilepsy syndrome characterized by focal or generalized febrile and afebrile seizures, and focal or generalized epileptiform discharges on EEG. The clinical presentation may differ considerably among affected individuals within the same family.^5,6^ Private mutations have been identified in genes encoding subunits of voltage-gated Na^+^ channels (*SCN1A*, *SCN1B*)^7,8^ and the γ-aminobutyric acid type A receptor (*GABRG2*, *GABRD*).^9,10^ Most of the genes that are associated with GGE and GEFS+ are also implicated in developmental and epileptic encephalopathies (DEEs), which are characterized by seizure onset in the first years of life, often pharmacoresistant seizures, cognitive regression, and neurologic deficits. For certain genes, such as *SCN1A*, a clear genotype-phenotype correlation is described: in GEFS+, a greater proportion of missense variants is found, whereas in Dravet syndrome, nonsense mutations or large deletions are more common.^11^

Recently, we reported variants in the *STX1B* gene as a cause of fever-associated epilepsies of variable severity.^12^ Two large families showed a rather benign course of GEFS+ syndrome, whereas 4 individuals had a more severe DEE phenotype. An additional 18-year-old patient exhibiting epilepsy with myoclonic-atonic seizures and moderate to severe intellectual disability was reported recently.^13^

*STX1B* encodes syntaxin-1B, a presynaptic protein that is part of the SNARE complex mediating the process of calcium-dependent synaptic vesicle release.^14^

We aimed to describe additional variants in *STX1B*, characterize the related clinical and EEG phenotypic spectrum, and establish genotype-phenotype correlations.

## Methods

### Clinical data

We identified 49 individuals harboring heterozygous variants in *STX1B* (NM_052874.3). The cases were pooled from 14 different centers identified through research studies and clinical diagnostic testing. The cohort consists of 7 families with 2 to 17 affected individuals and 16 sporadic patients. Of these, we previously reported 2 large families and 4 sporadic patients.^12,15,16^ We systematically collected clinical information such as age at onset, seizure type, neurologic and cognitive deficits, neuroimaging outcome, and antiepileptic drug (AED) treatment through a standardized questionnaire. We reviewed original EEG recordings (S. Wolking., H.L., T.D., C.M., P.W.-W., D.M.) for all but 3 patients (F3, F15, F18). EEGs for F1 and F2 were described in previous publications.^15,16^

### Standard protocol approvals, registrations, and patient consents

Written informed consent to participate in this study was obtained from all patients or caregivers.

### Genetics

We used different next-generation sequencing datasets from multicenter research projects or diagnostic testing services to identify the reported *STX1B* variants. The methods and the centers are available from Dryad (doi:10.5061/dryad.cf0hj73) and in our previous report.^12^ All patients are of European descent.

To evaluate the effect of the missense variants, we used well-established in silico prediction tools: SIFT (sorting tolerant from intolerant),^17^ PolyPhen-2 (polymorphism phenotyping v2),^18^ and MutationTaster.^19^ We compared patient *STX1B* missense variants to variants identified in almost 150,000 control individuals stored in the Genome Aggregation Database (gnomAD, gnomad.broadinstitute.org/).^20^ Mutation density was calculated by counting the number of variant positions in gnomAD divided by the window length within a window of 10 nucleotides using a sliding window approach over the coding sequence of the *STX1B* transcript NM_052874.3. We determined paralog conservation as described by Lal et al.^21^ In short, we aligned the canonical protein sequences of the 7 paralog genes of the Ensembl SYNTAXIN protein family, *STX2*, *STX1A*, *STX11*, *STX1B*, *STX19*, *STX3*, and *STX4* and scored the conservation at each alignment position using JalView.^22^ Then we determined the mean and the standard deviation paralog conservation for each single protein of the protein family and a *z* score (para_zscore) calculated for each residue position. We defined paralog conservation as para_zscore >0.

### Data availability

Data not published in this article will be shared as anonymized data by request from any qualified investigator.

## Results

### Phenotypic descriptions

Reviewing the clinical characteristics of all patients, we could distinguish 4 different phenotypic groups (numbering of the families was defined as follows: F1–F6 as in our previous study,^12^ F7–F23 new families sorted by phenotype). (1) Three families and 3 sporadic patients with FS, with or without additional generalized and more rarely focal afebrile seizures, with a relatively benign course, generally good drug response, normal development, and mild or no neuropsychiatric symptoms, corresponding to GEFS+ (F1, 2, 7–10). (2) Two unrelated patients with generalized myoclonic and absence seizures, without FS, and without major cognitive deficits, corresponding to juvenile myoclonic epilepsy (JME), a common subtype of GGE (F11, 12). (3) Thirteen unrelated patients with intractable seizures, occurrence of developmental stagnation or regression after seizure onset, and additional neuropsychiatric deficits compatible with DEE (F3–6, F13–21). (4) Two patients with some form of focal epilepsy (F22, 23). The main clinical characteristics of all families and sporadic patients are summarized in table 1. More detailed clinical data are available from Dryad, doi.org/10.5061/dryad.cf0hj73.

**Table 1:**
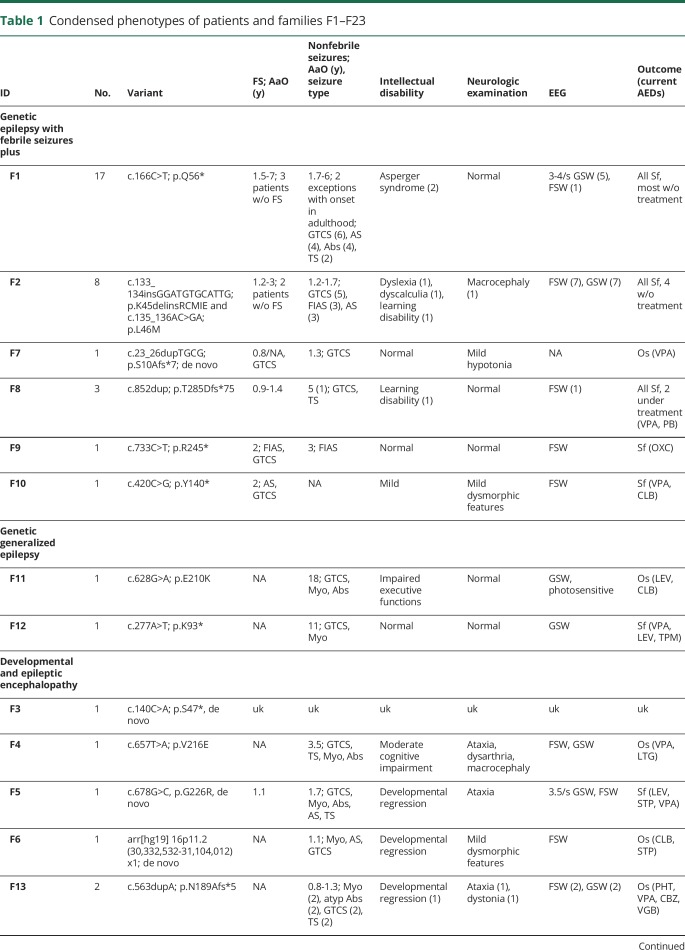

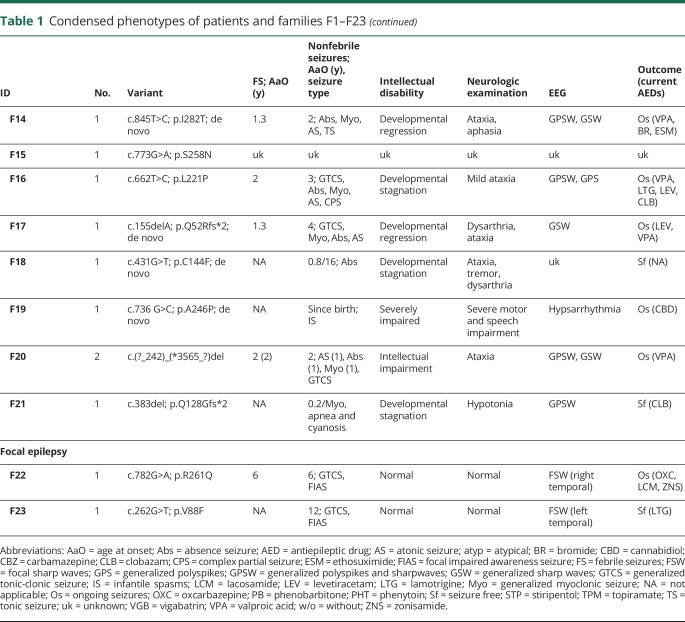
Condensed phenotypes of patients and families F1–F23

#### Group 1: GEFS+ (31 patients in families F1, F2, F7–10)

FS were present in the majority of patients. The first seizure started between 10 months and 5 years (median 20 months). There was a large variety of afebrile seizure types, including generalized tonic-clonic seizures (GTCS) (n = 13), focal impaired awareness seizures (n = 3), atonic seizures (n = 8), tonic seizures (n = 4), and absence seizures (n = 5). Nine patients had infrequent seizures until adulthood that did not require treatment. For most cases, cognition was intact and neurologic deficits were not frequent in this cohort (table 1). Asymptomatic variant carriers were identified in 2 families (4 in F1, 3 in F2), which is of importance for genetic testing and counseling in mild cases. Moreover, in F1, 4 phenocopies were identified (2 patients with a GGE phenotype not matching GEFS+, 1 individual with a single FS, and 1 patient with falls and head nodding in early childhood that were later interpreted as nonepileptic^15^).The treatment outcome in this group was overall positive. More than 50% of patients were seizure-free without medication (17); the remainder were seizure-free with AED treatment (11). Only patient F7 had ongoing seizures with valproic acid. EEGs showed focal and generalized epileptiform discharges. Available ictal recordings in F8.1 showed focal onset seizure.

#### Group 2: GGE (2 patients, F11, F12)

The first seizures for patient F11 were at age 18 years (generalized myoclonic seizures) followed by GTCS at 20 years, and absences at 21 years. GTCS occurred in clusters of 3 or 4 seizures every 3 months. On neuropsychological examination, the patient showed slight executive dysfunction; however, IQ and memory functions were normal. F12 had his first seizures at 11 years, featuring both afebrile myoclonic seizures and GTCS. A Wechsler Adult Intelligence test at 19 years revealed an IQ of 85 ± 3. On examination, neither patient showed deficits. Treatment was difficult, requiring multiple AED trials in both patients (table 1).

For F11, the first EEG at age 21 showed an unusual pattern of frequent bursts of generalized slow waves associated with myoclonic jerks of both shoulders. Later, typical generalized bursts of spikes and sharp waves were documented under photic stimulation (he was the only photosensitive patient in the whole cohort, as far as this was examined). For F12, the first available EEG was performed at age 19 and showed occasional brief bilateral epileptic discharges. A video-EEG at age 24 demonstrated 4-Hz generalized spike-wave activity. Video-EEG monitoring at age 31 showed no epileptic activity.

#### Group 3: DEE (15 patients, F3–6, F13–21)

We retrieved detailed phenotypes for 12 of 15 patients. Epilepsy onset was between 0 months and 3.5 years (median 15 months). Six patients (F5, F14, F16, F17, F20) had FS with onset between 13 months and 2 years. F14 had only a single FS after vaccination. Afebrile seizures comprised GTCS (n = 9), myoclonic (n = 10), atonic (n = 8), tonic seizures (n = 3), atypical absences (n = 3), infantile spasms (n = 1), and hyperkinetic focal seizures (n = 1).

Except for F6, initial development was normal in all patients, with severe global developmental delay or even regression coinciding with seizure onset. In F19 and F21, global developmental delay and seizures were present since birth. Neurologic examination showed anomalies in most patients with ataxia being the most frequent finding.

Seizures were pharmacoresistant in all patients except F18, undergoing on average 9 AED trials. Only F5 and F21 achieved seizure freedom with ongoing treatment. Of note, F4 responded with a significant seizure reduction to the combined therapy of lamotrigine and valproic acid after several unsuccessful AED trials. After the administration of bromide, F14 showed a significant reduction in seizure frequency.

EEGs were available for review in 11 of 15 patients. Interictally, the EEGs in all cases showed multifocal epileptic discharges that were predominantly located in the frontal or temporal region. In addition, we detected generalized discharges in 10 of 11 patients presenting as generalized spike-wave or polyspike-wave discharges. In 4 of 11 patients, EEGs showed frequent bursts of generalized rhythmic activity lasting several seconds. Ictal EEG recordings were available in 7 of 11 patients. In 4 patients, typical tonic seizures with generalized beta activity with increasing amplitude and decreasing frequency were recorded. EEG curves are available from Dryad (additional figures), doi:10.5061/dryad.cf0hj73.

#### Group 4: Focal epilepsy (2 patients, F22, F23)

F22 had right temporal lobe epilepsy, with a single FS reported at 6 years. Afebrile impaired awareness seizures with automatisms occurred at the same age. Sometimes she would describe an aura with a rushing sensation in her head or blurred vision. Occasionally, secondary generalization would occur. Interictal EEGs showed right temporal epileptiform discharges. Ictal recording of one seizure depicted a seizure onset over the entire right hemisphere, with subsequent evolution most prominent over the right temporal region. Neuropsychological testing and neurologic examination were unremarkable. Repeated MRIs showed nonspecific white matter lesions. An [^18^F]-fluorodeoxyglucose–PET at age 28 revealed hypometabolism in the right temporal lobe. Seizures were pharmacoresistant to oxcarbazepine, lacosamide, and zonisamide.

In F23, weekly seizures started in teenage years with staring episodes accompanied by subtle twitching of the right arm. In addition, GTCS started at 24 years. Seizures were controlled with lamotrigine. At 45 years, the patient developed stroke-like episodes featuring left-sided hemicrania, dilatation of the right pupil, hemihypesthesia, and mild hemiparesis. The episodes lasted between 30 and 120 minutes and occurred about once a week. An MRI at age 46 years showed no abnormalities. Previous EEGs showed left temporal epileptiform discharges. However, the most recent EEGs were normal. A video-EEG recorded during one of the stroke-like episodes was without EEG correlate. Of note, in the patient's fifth decade of life, several autoimmune diseases were diagnosed: type 1 diabetes mellitus, celiac disease, and hypogammaglobulinemia. Treatment with IV immunoglobulins led to a decrease in the frequency of the stroke-like episodes.

### Molecular genetics

**Figure 1:**
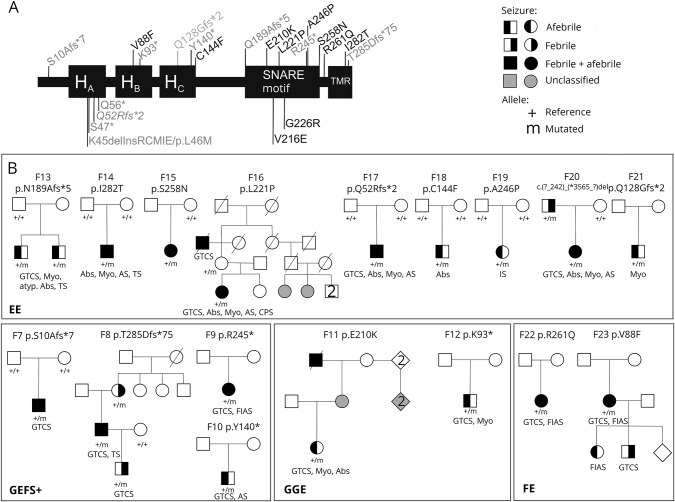
*STX1B* gene with variants and pedigrees of newly identified variants (A) Putative domain structure of syntaxin-1B derived from that for syntaxin-1A,^24^ as the isoforms share 83.6% of their amino acid sequences (using the alignment program ClustalO^39^). Shown are the functional domains and depiction of variants. Missense variants are colored in black, other variants are shown in gray. The boxed variants represent developmental and epileptic encephalopathies. (B) Pedigrees of sporadic patients/families with newly identified variants. F21 was adopted and there was no information about the biological parents. Abs = absence seizure; AS = atonic seizure; atyp. = atypical; CPS = complex partial seizure; FE = focal epilepsy; FIAS = focal impaired awareness seizure; GEFS+ = genetic epilepsies with febrile seizures plus; GGE = genetic generalized epilepsy; GTCS = generalized tonic-clonic seizure; IS = infantile spasms; Myo = generalized myoclonic seizure; TMR = transmembrane region; TS = tonic seizure.

**Figure 2:**
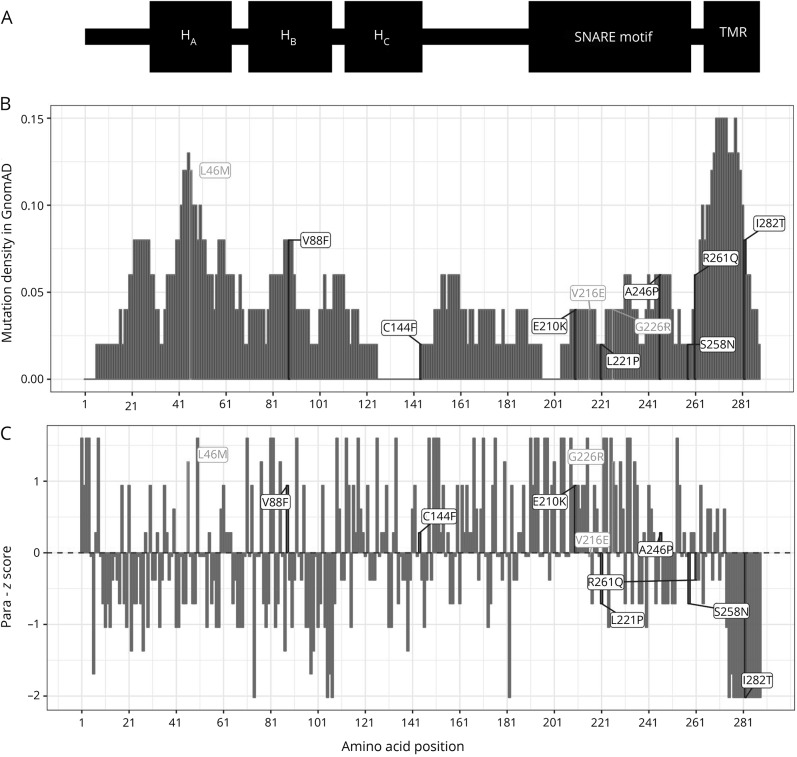
Mutational density and paralog conservation scores of *STX1B* missense variants (A) Putative domain structure of syntaxin-1B (figure 1A). (B) Variation density in *STX1B* amino acid positions according to the Genome Aggregation Database (gnomAD) depicting the reported missense variants. (C) Paralog conservation score (para_zscore) of the *STX1B* amino acid positions depicting the reported missense variants (positive values are considered as paralog conserved). Previously reported variants^12^ are colored in gray, new variants in black. TMR = transmembrane region.

**Figure 3:**
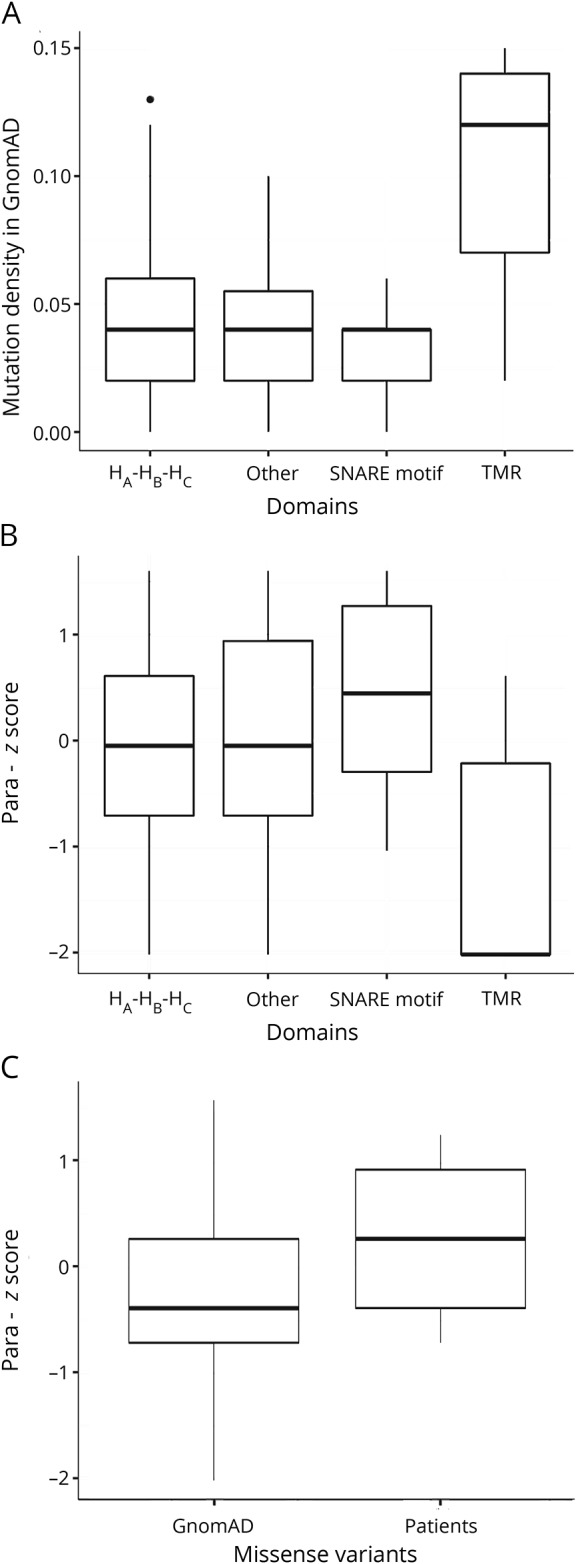
Mutational density and paralog conservation score of different gene regions (A) Distribution of mutational density of 4 *STX1B* gene regions. The SNARE motif shows the lowest mutational density, the transmembrane region (TMR) the highest density. (B) Paralog conservation score (para_zscore) distribution of 4 *STX1B* gene regions. The SNARE motif shows the highest paralog conservations, the TMR the lowest conservation. (C) Comparison of paralog conservation score for all Genome Aggregation Database (gnomAD) variants and patient variants.

Of the 17 newly identified variants in *STX1B*, 8 are missense (p.Val88Phe, p.Cys144Phe, p.Glu210Lys, p.Leu221Pro, p.Ala246Pro, p.Ser258Gln, p.Arg261Gln, p.Ile282Thr), 5 frameshift (p.Ser10Alafs*7, p.Gln52Argfs*2, p.Glu128Glyfs*2, p.Asn189Alafs*5, p.Thr285Aspfs*75), and 3 stop gain variants (p.Lys93*, p.Tyr140*, p.Arg245*). One patient had a deletion of the entire gene (F20). Syntaxin-1B consists of an N-terminal helical domain, containing the H_A_, H_B_, and H_C_ domains, the SNARE motif, and the C-terminal transmembrane domain (figure 1A). When in close proximity with SNAP-25 and synaptobrevin, syntaxin-1B forms the SNARE complex that catalyzes membrane fusion in Ca^2+^-triggered exocytosis. Syntaxin-1B can adopt 2 conformations: open and closed. Both conformations are important for exocytosis: the open conformation is necessary for the formation of the SNARE complex, whereas the closed conformation initiates the synaptic vesicle fusion reaction.^23,24^ Figure 1A shows the new variants combined with those previously reported in a schematic view of the syntaxin-1B protein. In comparison to the location of loss-of-function variants, missense variants were more frequent in the second part of the gene containing (1) the SNARE motif conveying interaction with the other 2 components of the SNARE complex and (2) the transmembrane domain, which is responsible for anchoring syntaxin-1B into the cell membrane. None of these variants was reported so far in the gnomAD database, currently the largest collection of exomes and genomes publicly available.^20^ In general, *STX1B* is intolerant to missense variants (missense *z* score >3.63) and haploinsufficiency (pLI score of 0.94) in the Exome Aggregation Consortium browser.^20^ Evaluation by different in silico prediction tools (SIFT,^17^ PolyPhen-2,^18^ MutationTaster^19^) showed a damaging effect for 6 of 8 variants in 3 of 3 prediction tools (table 2). We calculated the mutational density of missense variants in the general population from the Exome Aggregation Consortium using a sliding window approach and could show that the variants are located in regions of different mutational density (figure 2B). We also calculated a paralog conservation score distribution for *STX1B* (para_zscore)^21^ and highlighted the patient variants (figure 2C). This score gives the degree of conservation among paralog genes in the human Syntaxin gene family. Disease variants are enriched in paralog-conserved residues.^21^ In our paralog analysis, the SNARE motif turned out to be the most paralog-conserved and gnomAD variant–depleted region of the protein with the highest para_zscores (figure 3, A and B). Furthermore, in a direct comparison of patient and gnomAD missense variant distribution across the linear protein sequence, we observed a significant enrichment of patient variants in the SNARE motif (*p* = 0.036). We ascertained missense variants in an unbiased manner so that SNARE motif enrichment by chance is improbable. The amino acid positions of most of the detected missense variants showed high or intermediate para_zscores. There was no significant difference between the para_zscores from missense variant positions in patients compared to those in gnomAD (figure 3C). We also detected a few disease-associated variants in regions with a high mutational density in gnomAD and low para_zscores (p.Arg261Gln, p.Ile282Thr), which were located in the transmembrane domain or the part linking the SNARE motif with the transmembrane domain of the protein.

**Table 2:**
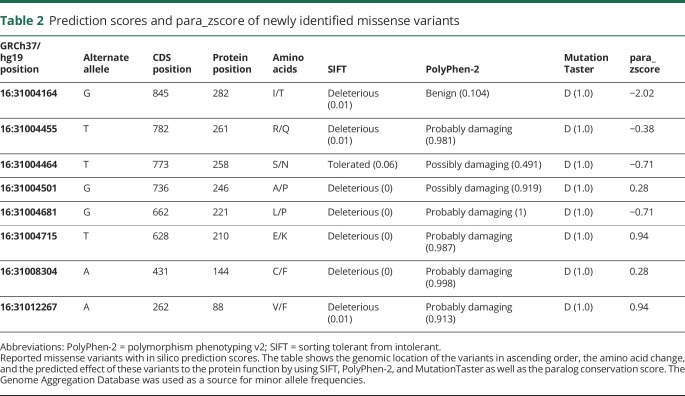
Prediction scores and para_zscore of newly identified missense variants

### Segregation of variants in families and family histories

The segregation status for all variants in the respective pedigrees, together with a classification of the phenotypes, is shown in figure 1B. Previously published pedigrees can be found in Schubert et al*.*^12^ We validated inheritance in 6 of the total 23 variants in families with more than one affected (4 new and 2 published). They were mostly cosegregating with their phenotypes but also included both asymptomatic variant carriers and phenocopies (F1, 2, 8, 13, 16, 20). In 3 patients, a positive family history of epilepsy was available, but additional family members were not available for genetic testing (F4, 11, 23). The available phenotypes of the other affected individuals are depicted in figure 1B.

We showed de novo occurrence for 8 of the remaining 14 variants, which were all detected in sporadic patients (F3, 5, 6, 7, 14, 17, 18, 19), whereas in 6 patients, the respective family members were not available for segregation analysis (F9, 10, 12, 15, 21, 22).

## Discussion

The current study expands the number of reported patients with *STX1B*-related epileptic syndromes and describes 17 new variants. Four different phenotypes can be discerned: (1) a benign epilepsy syndrome with febrile and afebrile seizures corresponding to GEFS+, (2) a GGE phenotype, (3) a DEE syndrome with refractory seizures and moderate to severe developmental deficits, and (4) a focal epilepsy phenotype. *STX1B*-related epilepsies thus show a remarkable phenotypic heterogeneity that is in its extent reminiscent of other epilepsy-related genes, such as *SCN1A*,^11^ *SCN2A*,^25^ *KCNQ2*,^26^ and *STXBP1*.^27^ In comparison to *STXBP1*, which is closely related to *STX1B* (since the respective proteins interact with each other in the synaptic transmitter release machinery) and exhibits in most cases an early onset of epilepsy presenting as Ohtahara syndrome or West syndrome,^28^ *STX1B-*related DEE shows a later onset of epilepsy after the first birthday. Exceptions to this rule are F19 (p.A246P) and F21 (p.Q128Gfs*2) who presented with a seizure onset within the first days of life and are more reminiscent of *STXBP1*-related syndromes. It is of interest that *STX1B-* and *STXBP1-*related DEEs both feature ataxia and other movement disorders.^28^ However, in the vast majority of *STXBP1*-related epilepsy syndromes, moderate to severe developmental delay is present^27–29^—milder phenotypes, as in our cohort, are not observed. In comparison to other common DEE disorders, the median time of seizure onset differentiates: neonatal or early infantile for *SCN2A* and *KCNQ2* (a later onset with these genes is rarer and associated with different functional consequences—gain- vs loss-of-function mutations^25,26,30^) and later infantile for *SCN1A* and *STX1B*. Also in other aspects, parallels can be drawn with *SCN1A*-related disorders. Thus, in 5 *STX1B*-related DEE cases, FS and the presence of myoclonic seizures were reminiscent of Dravet syndrome.^31^ However, the presence of tonic seizures in these patients would be unusual for Dravet syndrome. A patient with generalized tonic-clonic, atonic, and myoclonic seizures with onset at 1 year and severe developmental delay was reported before this study with a 1.2 Mb de novo 16p11.2 deletion,^13^ including the *STX1B* gene. The phenotype was classified as epilepsy with myoclonic-atonic seizures, a syndrome that has been associated with *SCN1A* in several patients.^32^ The previously reported patient showed a remarkable resemblance to F6 and F20, who also harbor a comparable deletion. Another similarity with *SCN1A* is that our cohort features several patients and families with GEFS+ syndrome^7^ and one patient with mesial temporal lobe epilepsy and FS (for which common polymorphisms in *SCN1A* have been described as a genetic risk factor^33^), and at least one GEFS+ multiplex family with mesial temporal lobe epilepsy has been reported.^34^ Of interest is our patient with episodes reminiscent of hemiplegic migraine; *SCN1A* mutations are a recognized cause of familial hemiplegic migraine.^35^ We acknowledge that the association with a migraine-like syndrome in a single patient could also be coincidental. In contrast, classic GGE syndromes, such as represented by the 2 patients with JME in our cohort, are not typically described in patients with *SCN1A* mutations. Of note, both patients with JME had an atypical response of pharmacoresistant seizures. In the DEE group, 7 of 13 (F3, F5, F6, F14, F17, F18, F19) variants were de novo*.* This supports the likelihood of a causative role in the context of the observed phenotypes. Three missense variants showed a high paralog conservation (p.C144F, p.G226R, p.A246P), increasing the probability of a functional role. An exception to this rule was p.I282T (F14), a variant that was discovered using a gene panel approach. Possibly, despite the de novo status of this variant, another as yet unknown genetic alteration in a gene not captured by the gene panel might be more relevant in this case. Although variant p.V216E (F4) showed a slightly negative paralog score, this variant lies in a highly conserved region. The patient had a positive family history and previous functional data, i.e., rescue experiments in *STX1B* knockdown zebra fish larvae^12^ support the pathogenicity of this variant. In F13 (p.N189Afs*5), both affected siblings had a similar phenotype. This suggests an inheritance from the mother, who has not been tested, while the father was tested negative. Since the mother and other maternal family members were reportedly not affected, germline mosaicism could be possible. However, other genetic causes cannot be entirely excluded because the variant was found in a gene panel of 85 epilepsy genes. In F15 (p.S258N), the prediction tools suggest a benign variant. Since no family members were tested and the paralog score was rather low, the role of this variant has to be considered carefully. However, the variant is located at the end of the SNARE motif, which supports a causative role. In F16 (p.L221P), the family history suggests an inherited defect. The variant was identified by exome sequencing and no other convincing variants were found. The unaffected mother also carries the variant but other affected family members were not available for testing. Prediction tools indicate a deleterious variant and the variant is located in the middle of the SNARE motif, although the paralog score was rather low; a case of incomplete penetrance is therefore possible or the variant could represent a predisposing factor for epilepsy. In F20, the gene deletion was inherited from the father, who had a milder phenotype, suggesting the presence of other genetic modifiers. In F21 (p.Q128Gfs*2), the parents were not tested and the variant was identified in a gene panel of 115 candidate genes, so that other genetic causes cannot be excluded. In the GEFS+ group, in 3 of 6 cases (F1, F2, F8), a positive family history with cosegregating genetic findings support the causative role of the reported variants. F7 (p.S10Afs7) was confirmed to be de novo*.* For F9 (p.R245*) and F10 (p.Y140*), the parents were not tested. Since all variants in this group predict a complete loss of function, we consider them as disease-causing and the mechanism for the GEFS+ group seems to be clearly a haploinsufficiency. In the GGE group, neither case had parents who were tested. F11 (p.E210K) has a positive family history that is suggestive of an inherited disease. The causative role of this variant is underscored by the in silico findings that the variant is predicted deleterious, lies in a region of very low mutational density, and shows high paralog conservation. p.K93* (F12) leads to a haploinsufficiency, in line with the variants causing GEFS+. Furthermore, this variant was identified by whole-genome sequencing and no other convincing variants were found. Of interest, in a recent genome-wide association study of common epilepsies, a significant signal in the region of *STX1B* was detected in the JME cohort.^36^ In the FE group, the role of *STX1B* should be considered more carefully. In F22 (p.R261Q) and F23 (p.V88F), parents were not tested. For p.R261Q, the established prediction tools point toward a deleterious variant, while the position shows medium mutational density and low paralog conservation. However, whole-genome sequencing showed no other likely pathogenic variants. For p.V88F, the used prediction tools show a more coherent picture of a deleterious variant, the paralog score was high, and whole-exome sequencing found no other convincing variants.

Regarding genotype-phenotype correlation, it is notable that missense variants in the functionally relevant SNARE motif were identified in 6 of 7 patients or families (86%) associated with DEE. The seventh patient (p.E210K) had refractory JME. In contrast, all but one of the GEFS+ patients or families had truncating variants (5 of 6, 83%) predicting nonsense-mediated decay and complete loss of function of one allele indicating haploinsufficiency as the disease mechanism.

A possible explanation could be that missense variants in crucial protein regions, such as the SNARE motif, may lead to a deficient gene product that interferes with protein-protein interactions and normal presynaptic vesicle fusion (dominant-negative effect), whereas missense variants in the first part of the gene may affect assembly of syntaxin-1B proteins and therefore have similar consequences as a truncation with nonsense-mediated decay. The loss-of-function mechanisms could also be compensated—at least in part—by syntaxin-1A and, thus, be less damaging.^37^

We demonstrate that *STX1B* variants are linked to 4 different epilepsy phenotypes. De novo dominant mutagenesis is the etiology of most DEEs, whereas the more common epilepsies are likely complex genetic disorders. This work suggests a role for ultrarare *STX1B* variants in the pathogenesis of a broad range of both common and rare epilepsies. There are plausible explanations for genotype-phenotype correlations, such that haploinsufficiency causes rather benign phenotypes, whereas missense variants in functionally critical regions cause more severe phenotypes. Other, unknown factors, which may include genetic modifier, individual genetic background, and epigenetic effects, could also have an important role and explain phenotypic heterogeneity, reduced penetrance, and variable phenotypes among carriers of truncating mutations. Functional studies on a protein and cellular neuronal level will shed more light on the relevance and degree of severity of the described variants and contribute to a better understanding of the dysfunction of syntaxin-1B and synaptic transmission in epilepsy and strengthen our current interpretation of genotype-phenotype correlations.

## Glossary

DEE: developmental and epileptic encephalopathy
FS: febrile seizures
GEFS+: genetic epilepsies with febrile seizures plus
GGE: genetic generalized epilepsy
gnomAD: Genome Aggregation Database
GTCS: generalized tonic-clonic seizure
JME: juvenile myoclonic epilepsy
PolyPhen-2: polymorphism phenotyping v2
SIFT: sorting tolerant from intolerant
SNARE: SNAP (soluble NSF attachment protein) receptor
